# Decreasing Risk of Fatal Subarachnoid Hemorrhage and Other Epidemiological Trends in the Era of Coiling Implementation in Australia

**DOI:** 10.3389/fneur.2017.00424

**Published:** 2017-08-31

**Authors:** John Mark Worthington, Chris Goumas, Bin Jalaludin, Melina Gattellari

**Affiliations:** ^1^Institute of Clinical Neurosciences, Royal Prince Alfred Hospital, Sydney, NSW, Australia; ^2^South Western Sydney Clinical School UNSW, Liverpool, NSW, Australia; ^3^Ingham Institute for Applied Medical Research, Liverpool, NSW, Australia; ^4^School of Public Health and Community Medicine, University of New South Wales, Sydney, NSW, Australia

**Keywords:** subarachnoid hemorrhage, epidemiology, time trends, socio-economic status, region of birth, fatality

## Abstract

**Background and purpose:**

Subarachnoid hemorrhage (SAH) is associated with a high risk of mortality and disability in survivors. We examined the epidemiology and burden of SAH in our population during a time services were re-organized to facilitate access to evidence-based endovascular coiling and neurosurgical care.

**Methods:**

SAH hospitalizations from 2001 to 2009, in New South Wales, Australia, were linked to death registrations to June 30, 2010. We assessed the variability of admission rates, fatal SAH rates and case fatality over time and according to patient demographic characteristics.

**Results:**

There were 4,945 eligible patients admitted to hospital with SAH. The risk of fatal SAH significantly decreased by 2.7% on average per year (95% CI = 0.3–4.9%). Case fatality at 2, 30, 90, and 365 days significantly declined over time. The average annual percentage reduction in mortality ranged from 4.4% for 30-day mortality (95% CI −6.1 to −2.7) (*P* < 0.001) to 4.7% for mortality within 2 days (−7.1 to −2.2) (*P* < 0.001) (Table 3). Three percent of patients received coiling at the start of the study period, increasing to 28% at the end (*P*-value for trend <0.001). Females were significantly more likely to be hospitalized for a SAH compared to males [incident rate ratio (IRR) = 1.33, 95% CI = 1.23–1.44] (*P* < 0.001) and to die from SAH (IRR = 1.40, 95% CI = 1.24–1.59) (*P* < 0.001). People born in South-East Asia and the Oceania region had a significantly increased risk of SAH, while the risk of fatal SAH was greater in South-East and North-East Asian born residents. People residing in areas of least disadvantage had the lowest risk of hospitalization (IRR = 0.83, 95% CI = 0.74–0.92) and also the lowest risk of fatal SAH (0.81, 95% CI = 0.66–1.00) (*P* < 0.001 and *P* = 0.003, respectively). For every 100 SAH admissions, 20 and 15 might be avoided in males and females, respectively, if the risk of SAH in our population equated to that of the most socio-economically advantaged.

**Conclusion:**

Our study reports reductions in mortality risk in SAH corresponding to identifiable changes in health service delivery and evolving treatments such as coiling. Addressing inequities in SAH risk and mortality may require the targeting of prevalent and modifiable risk factors to improve population outcomes.

## Introduction

Subarachnoid hemorrhage (SAH) is the most devastating form of stroke due to its high risk of early mortality, long-term disability in survivors, and relatively young age at onset (1, 2). Ruptured aneurysms and arteriovenous malformations cause SAH in the majority of cases, while conventional stroke risk factors such as hypertension and poorly controlled antithrombotic medications will predispose a substantial minority of generally older people to a SAH (3).

Modern management of SAH involves urgent neurosurgical care to secure ruptured aneurysms and arteriovenous malformations to prevent catastrophic rebleeding. Rapid assessment of patients with suspected SAH, the upscaling of emergency services to expedite the diagnosis and transfer of patients, and around the clock access to expert neurosurgeons and interventional radiologists are cornerstones of modern management. In cases not otherwise suitable for definitive interventions, management in specialist neurosurgical centers improves outcomes (4).

In 2005, the landmark ISAT study established coiling as first-line treatment for selected aneurysmal SAH cases (5). In our state-wide health service, an existing network of 10 neurosurgical units located in tertiary referral centers provided organized services, prioritizing the early transfer and intensive neurocritical care of SAH patients. Funding for the establishment or enhancement of stroke units occurred in 2003 and in 2006 neurointerventional networks was established to improve access to coiling treatment.

Studies utilizing multiple overlapping sources of cases and independent standardized validation of clinical diagnoses are considered hallmarks of gold standard epidemiological methods. However, such studies are typically labor intensive and have yielded small numbers of SAH cases, limiting the capacity to examine trends over time and the impact of emerging endovascular treatment on patient outcomes (1, 2). Administrative data offer an alternative source of data, which may be harnessed to study the epidemiology and outcomes of SAH. The advantage of routinely collected data is that a large number of cases can be retrospectively identified for a population defined in geographical location and time, simulating population-based selection of a representative cohort of cases. In Australia, routinely collected hospitalization data provide a continuous census of all public and private hospital admissions. As such, these represent a population-based source of hospitalized cases and offer an opportunity to cost-effectively study time trends in admission rates and the impact of health services on patient care and outcomes. Linkage of hospitalization data to death registries allows researchers to ascertain mortality rates in the acute and post-acute periods and in the long term after patients have left hospital with high rates of follow-up. For an uncommon condition, such as SAH, administrative data therefore provide a means for a large-scale population-based study, which would otherwise not be feasible allowing comparisons of risk and mortality due to socio-demographic factors, such as socio-economic status (SES) and region of birth.

We undertook an analysis of administrative health data to examine the burden of SAH in our health service and to explore trends in SAH risk and mortality during a time of significant restructuring of neurocritical services. We also determined the variability in the burden of SAH due to socio-demographic factors.

## Materials and Methods

### Patient Selection

Acute cases of SAH were identified from the Admitted Patient Data Collection from January 1, 2001, to December 31, 2009, and were linked to mortality data to June 30, 2010. These data represented all available data at the time of extraction. The study period coincided with the publication of preliminary data from the ISAT trial in 2002 (6) and provided approximately an equal number of years before and after the final ISAT results that were published in October 2005 (5). The context in which we examined time trends, therefore, is described as the era during which coiling emerged as an evidence-based therapy providing the most relevant time period during which to determine the effects of the targeted health service restructuring in direct response to this new procedure.

The analysis was carried out as part of a larger study exploring the epidemiology and outcomes of ischemic stroke (7), transient ischemic attack (8), and intracerebral hemorrhage (9). Data linkage was carried out by the Center for Health Record linkage, a government-funded provider of linkage services for researchers and policy makers. Deterministic and probabilistic linkages using patient identifiers, enhanced by manual review, were used to create individual level data linking hospital and mortality datasets. Ethically approved gold standard privacy-preserving protocols enabled data to be linked and then released to researchers with patient identifiers removed. False-positive and false-negative links are estimated to be no greater than 5 per 1,000 (10).

The Admitted Patient Data Collection is a census of all hospitalizations in New South Wales, Australia’s most populous state (~7 million and 33% of the resident population) (11), and includes deaths in emergency departments (12). We selected principal diagnoses of SAH using the International Classification of Diseases version 10 Australian Modification (ICD-10-AM) (13) (I60.x), excluding cases residing outside New South Wales and those discharged within 48 h consistent with previous research (14) or with a concomitant diagnosis of traumatic head injury or stroke sequelae as likely misclassified cases. Acute secondary diagnoses of SAH were included if the principal diagnosis was consistent with an acute SAH (for example, if a diagnosis of cardiac arrhythmia was recorded) or excluded if inconsistent (for example, post-operative review).

### SAH Admission Rates, Fatal Rates, and Case Fatality

We calculated crude admission rates per 100,000 person years using denominators derived from population data for each year of our study period (15). Rates were directly standardized to the World population to produce age-standardized rates (ASRs) (16). Only cases aged 20 years or older were included for these analyses as data for variables of interest were provided in 5 year age groups. We included both first (index) and recurrent SAH admissions. Hospital admissions were linked to the state death registry to June 30, 2010. Fatal SAH defined as death occurring within 30 days of admission, provided a population-based measure of the impact of hospital management on SAH survival necessarily excluding cases not reaching hospital. Cause of death data were available until 2007, and we conducted a sensitivity analysis including “sudden deaths” to estimate the risk of SAH irrespective of hospital admission. Crude case fatality rates were calculated for 2, 7, 30, 90, and 365 days by dividing the number of deaths within each time period by the number of SAH cases. For the analysis of case fatality, index admissions for patients aged 18 years or older were utilized. Case fatality estimates for 365 days was available for all cases to June 30, 2009, and thus, cases recorded during the second half of 2009 were excluded from that analysis.

### Time Trends

The average annual percentage change (APC) in relative terms was calculated over the study period using negative binomial regression models. The outcome equaled the number of SAH admissions offset against the natural logarithm of the age- and sex-specific population. Year of diagnosis was included in models as a continuous linear predictor variable. Age- and sex-adjusted trends for the total population and by sex and age group were estimated. Two-way interaction terms between age, sex, and year of diagnosis were tested. The APC over time in mortality at 2, 7, 30, and 365 days was estimated using Cox regression models adjusted for age and sex.

### Uptake of Coiling

We interrogated procedure coding as applied elsewhere (17) to identify cases undergoing endovascular coiling.

### Variability in SAH Rates

Negative binomial models estimating Incident Rate Ratios (IRRs) determined the variability in SAH rates according to age and sex. Age- and sex-adjusted comparisons according to SES, region of birth (18), and geographic accessibility to community and health services were also carried out. The Index of Relative Socio-Economic Disadvantage (IRSD) (1) and the Accessibility/Remoteness Index of Australia (19), standardized measures of SES deprivation, and geographic remoteness were derived from patient residence within geographical distinct territories known as Local Government Areas. A mandatory national census of all residents is carried out once every 5 years recording proxy measures of SES with over 95% participation of households (20). Variables used to inform SES included educational attainment, occupation, income, English fluency, rental accommodation, and car ownership (21). IRSD scores are created using quintile cutoffs where a score of 1 indicates residence in areas of the greatest socio-economic disadvantage (that is, relatively less affluent) and a score of 5 indicates residence in areas of least socio-economic disadvantage (that is, relatively more affluent) (21).

We calculated the excess rate of SAH attributable to socio-economic deprivation. Using crude and age adjusted rates, the following formula was applied: population attributable risk percentage = [(*I_r_*−*I*_0_)/*I_r_*] × 100, where *I_r_* = incidence of SAH admissions in the population and *I*_0_ = incidence of overall SAH in the population residing in the least socio-economic disadvantaged areas (non-exposed group).

### Ethics Approval

The study was carried out in accordance with approvals provided by the New South Wales Population and Health Services Research Ethics Committee and the University of New South Wales. As the study utilized routinely collected data from which patient identifiers were removed, a consent waiver was granted.

## Results

### Patient Selection

Between 2001 and 2009, we identified 5,634 hospitalized cases of acute SAH, excluding 232 (4.1%) non-New South Wales residents, 160 (2.8%) discharged alive within 48 h, 193 and 38 (3.4 and 0.7%) with a concomitant code for traumatic head injury and stroke sequelae, respectively, and 66 (1.2%) with a disqualifying principal diagnosis. Of the 4,945 eligible patients (87.8%), 59.8% (*N* = 2,956) were female with a median age of 58 years [interquartile range (IQR) = 47–74 years]; males had a median age of 55 years (IQR = 45–69 years). Just under 30% (*N* = 1,428; 28.8%) were born overseas. Over 90% (*N* = 4,609, 93.2%) of cases had a principal SAH diagnosis recorded.

### Trend in Admission Rates

Crude hospitalization rates for females was 12.9 per 100,000 person years and 9.0 per 100,000 person years for males. Age-standardized rates were 10.7 and 8.1 per 100,000, respectively, for females and males (Table 1). Therefore, women were 1.32 times more likely to be hospitalized for SAH. Confidence intervals for age-standardized rates for males and females aged 20–29 years overlapped suggesting similar risk between the sexes in this age group. Age-standardized rates were higher for women than men in those aged over 30 years, with non-overlapping confidence limits for men and women aged over 40 (Table 1).

**Table 1:** Age-standardized rates^a^ (ASR) of subarachnoid hemorrhage (SAH) per 100,000 persons in New South Wales, Australia.

	Admission rates				Fatal SAH rates			
Age group	SAH cases	Crude rate	WHO ASR	ASR 95% CI	SAH cases	Crude rate	WHO ASR	ASR 95% CI
**Males**								
20–29	103	2.4	2.4	2.0–2.9	12	0.3	0.3	0.1–0.5
30–39	201	4.5	4.5	3.9–5.1	22	0.5	0.5	0.3–0.7
40–49	429	9.8	9.7	8.8–10.7	73	1.7	1.6	1.3–2.0
50–59	451	12.0	12.0	10.9–13.1	93	2.5	2.5	2.0–3.0
60–69	331	12.7	12.7	11.4–14.1	69	2.7	2.7	2.1–3.3
70–79	266	15.5	15.4	13.6–17.3	121	7.1	7.0	5.8–8.3
80–89	186	25.7	25.7	22.1–29.5	90	12.4	12.5	10.0–15.2
90+	22	25.9	25.9	16.1–37.9	16	18.8	19.3	10.9–30.0

Total	1,989	9.0	8.1	7.7–8.4	496	2.3	1.9	1.7–2.0

**Females**								
20–29	95	2.3	2.2	1.8–2.7				
30–39	261	5.8	5.7	5.0–6.4	40	0.5	0.4	0.3–0.6^b^
40–49	555	12.5	12.4	11.4–13.5	94	2.1	2.1	1.7–2.5
50–59	634	16.8	16.8	15.5–18.1	99	2.6	2.6	2.1–3.2
60–69	470	17.8	17.8	16.2–19.5	113	4.3	4.3	3.5–5.1
70–79	481	24.3	24.0	21.9–26.3	218	11.0	10.8	9.4–12.2
80–89	390	33.6	33.2	29.9–36.5	236	20.3	19.9	17.5–22.6
90+	70	29.7	29.7	23.1–37.0	56	23.8	23.7	17.9–30.4

Total	2,956	12.9	10.7	10.3–11.2	856	3.7	2.5	2.4–2.7

*^a^Rates standardized according to the World Health Organization (WHO) standard population*.

*^b^Fatal rate for females aged 20–39 years of age*.

Age- and sex-adjusted hospital admission rates remained unchanged over the study period and were stable for both sexes. However, time trends varied according to age (*P*_interaction_ = 0.02), declining an average of 3.0% (95% CI = 0.4–5.5%) for those aged between 20 and 49 years of age, remaining static for 50- and 69-year olds (APC = 0.6%, 95% CI = −1.3 to 2.4%) and increasing slightly for people older than 70 years (APC = 1.7; 95% CI = −0.6 to 4.1%). When hospitalization rates by age were examined according to sex, changes over time varied in males only (*P*_interaction_ = 0.007) with a significant decline seen only in males aged 20–49 years of age (APC = −4.6%, 95% CI = −7.9 to −1.2%) (*P* = 0.009) (Table 2).

**Table 2:** Average annual percentage change (APC) in subarachnoid hemorrhage (SAH) rates in New South Wales, Australia 2001–2009.

	Overall admission rates Attack rates				Fatal SAH rates 30-days		
	APC (95% CI)	*P*-value	*P*-value interaction‡		APC (95% CI)	*P*-value	*P*-value interaction‡
All cases*	−0.2 (−1.5 to 1.1)	0.73		All cases*	−2.7 (−4.9 to −0.3)	0.03	–
Sex^a^			0.46	Sex^a^			0.26
Males	−0.7 (−2.6 to 1.2)	0.45		Males	−4.1 (−7.3 to 0.7)	0.02	
Females	0.2 (−1.5 to 2.0)	0.82		Females	−1.6 (−4.7 to 1.5)	0.3	
Age^b^			0.02	Age^b^			0.03
20–49	−3.0 (−5.5 to 0.4)	0.02		20–49	−6.9 (−11.8–1.8)	0.008	
50–69	0.6 (−1.3 to 2.4)	0.55		50–69	−4.7 (−8.5 to 0.6)	0.02	
70+	1.7 (−0.6 to 4.1)	0.16		70+	0.4 (−2.9 to 3.8)	0.82	

*Age and sex adjusted.

*‡P-Value for interaction with year of admission*.

*^a^Age adjusted*.

*^b^Adjusted for sex*.

### Trends in Fatal SAH

Within 30 days, 1,352 of the 4,945 of hospitalized SAH cases had died (27.3%). Rates of fatal SAH were higher in females compared with males (Table 2). The risk of fatal hospitalized SAH cases significantly decreased by an average of 2.7% per year over the study period (95% CI = 0.3–4.9%) (*P* = 0.03). The change was greater for males than females (APC = −4.1%, 95% CI = −7.3 to −0.7% versus −1.6%, 95% CI = −4.7 to 1.5%), although the interaction term between sex and year was not statistically significant (*P*_interaction_ = 0.26), suggesting no difference between males and females in their risk of fatal SAH over time.

The decline in mortality was modified by age (*P*_interaction_ = 0.03). For 20–49-year olds, the average annual percentage decline in mortality was 6.9% (95% CI = 1.8–11.8%), while SAH mortality decreased by an average of 4.7% in relative terms in those aged between 50 and 69 years (95% CI = 0.6–8.5%). However, rates were stable in people over 70 years of age (APC = 0.4%, 95% CI = −2.9 to 3.8%). The APC by age for males and females was consistent with this pattern of a greater decline in mortality rates for younger compared with older patients.

### Trends in Case Fatality Rate

Within 2 days of hospital admission, 14.7% of SAH patients died. By 7 and 30 days, 21.5% and 28.7%, respectively, had died. Mortality thereafter increased marginally with 30.7% and just under one-third of cases dying within 90 and 365 days, respectively (Table 3). Case fatality at all measured time points significantly declined over time. The average percentage reduction in mortality ranged from 4.4% for 30-day mortality (95% CI −6.1 to −2.7) (*P* < 0.001) to 4.7% for mortality within 2 days (−7.1 to −2.2) (*P* < 0.001) (Table 3). At the beginning of the study period, 16% of patients (age- and sex-adjusted proportions) died within 2 days of hospital admission and 12% by its end, while 30-day mortality reduced from 26.5 to 22.2%. There was no evidence case fatality risk varied due to age or sex (*P*_interaction_=0.16–0.93). Around one in five SAH patients underwent coiling in the first 4 years of the study period, increasing to 28% in the second half of the study period (Table 3).

**Table 3:** Age- and sex-adjusted case fatalities (%).

Year	2 days	7 days	30 days	90 days	365 days	Percentage coiled^a,b^ (%)
2001	15.1 (12.3–17.8)	20.2 (16.6–23.7)	26.5 (23.0–29.9)	28.5 (25.1–31.7)	30.9 (27.2–34.3)	3
2002	16.0 (12.8–19.0)	24.0 (20.4–27.4)	30.7 (26.7–34.5)	32.8 (28.8–36.6)	35.1 (31.1–38.8)	10
2003	16.3 (14.1–18.3)	22.0 (19.5–24.5)	28.6 (24.9–32.1)	31.2 (27.4–34.8)	32.9 (29.1–36.6)	21
2004	14.3 (11.0–17.4)	20.5 (16.4–24.4)	27.3 (23.0–31.4)	28.9 (24.8–32.7)	30.6 (26.3–34.6)	22
2005	13.6 (10.9–16.2)	18.3 (15.3–21.3)	25.3 (21.9–28.5)	27.1 (23.7–30.4)	28.6 (24.9–32.2)	26
2006	12.8 (10.2–15.3)	19.8 (16.7–22.7)	26.5 (23.5–29.4)	27.8 (25.0–30.6)	29.1 (25.9–32.1)	33
2007	12.1 (9.3–14.8)	17.9 (14.6–21.1)	23.5 (20.1–26.8)	25.6 (22.2–28.9)	28.0 (24.8–31.1)	30
2008	11.4 (8.6–14.1)	15.8 (12.4–19.0)	21.0 (17.0–24.7)	22.1 (18.3–25.7)	23.9 (20.2–27.3)	30
2009	12.0 (9.2–14.7)	16.7 (13.2–20.0)	22.2 (18.7–25.5)	23.7 (20.1–27.2)	24.9 (21.3–28.2)	28
2001–2009	13.6 (12.2–14.9)	19.3 (17.5–21.0)	25.5 (23.6–27.4)	27.3 (25.4–29.2)	29.1 (27.2–31.0)	
Annual percentage change (95% CI)	−4.7 (−7.1 to −2.2)	−4.6 (−6.6 to −2.5)	−4.4 (−6.1 to −2.7)	−4.6 (−6.2 to −3.0)	−4.5 (−6.1 to −3.0)	
Deaths	697	1,011	1,341	1,430	1,445	
Total *N*	4,604	4,604	4,604	4,604	4,331	
Crude fatality	14.7 (13.3–16.1)	21.5 (19.7–23.3)	28.7 (26.6–30.7)	30.7 (28.6–32.7)	32.6 (30.5–34.7)	

*^a^Cox regression models adjusted for age, quadratic age, and sex; Ps < 0.01*.

*^b^P-Value for trend <0.0001*.

The proportion of cases receiving coiling significantly increased over the study period (*P*-value_trend_ = <0.0001). During the first year, 2001, only 3% of cases underwent coiling increasing to 10% during the second year (2002), with the proportion doubling in the following 12 months to 21%. The proportion of cases coiled peaked in 2006 to 33%. By the end of the study period, 28% of cases had been coiled (Table 3), representing an eightfold increase in the number of cases receiving coiling.

### Variability in Hospital Admission Rates by Patient Characteristics

**Figure 1:**
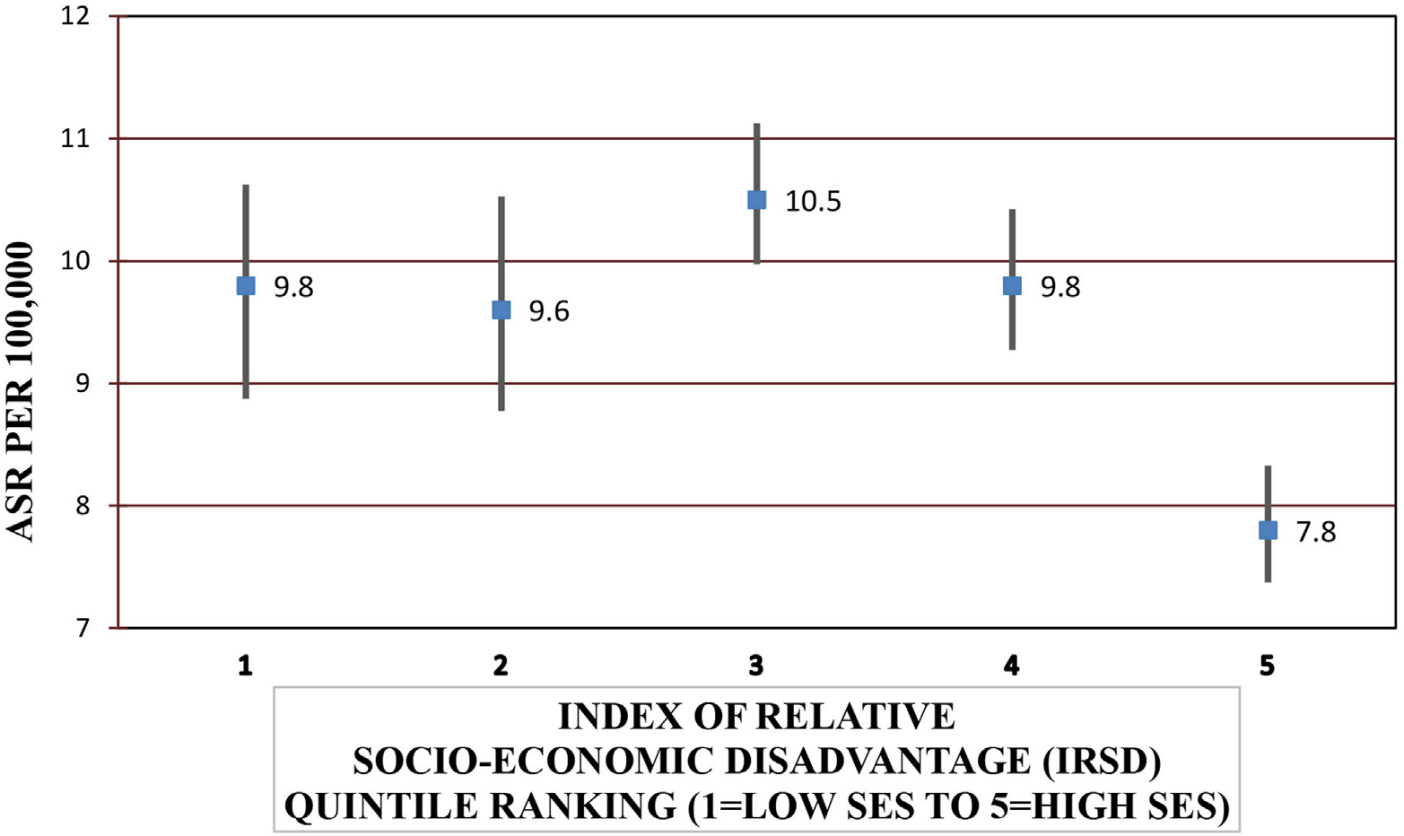
Age-standardized rates (ASRs) for subarachnoid hemorrhage admissions according to socio-economic status (SES).*Excludes 27 cases with missing local government area of residence.

Females were significantly more likely to be hospitalized for a SAH compared with males [IRR = 1.33, 95% CI = 1.23–1.44] (*P* < 0.001) and to die from SAH (IRR = 1.40, 95% CI = 1.24–1.59) (*P* < 0.001). Increasing age increased the risk of SAH admission and fatal SAH (*P* < 0.001) (Table 4). SES was significantly associated with SAH admission and mortality after hospitalization (*P* < 0.001 and *P* = 0.003, respectively) (Figure 1). People residing in areas of least disadvantage had the lowest risk of hospitalization (IRR = 0.83, 95% CI = 0.74–0.92) and also the lowest risk of fatal SAH (0.81, 95% CI = 0.66–1.00). Their age-standardized admission rate was 7.8 (95% CI = 7.4–8.3) per 100,000 persons, while the rate varied from 9.6 to 10.5 per 100,000 for other quintile bands representing greater relative socio-economic disadvantage. This effect of SES was evident in patients younger than 70 years of age (*P*_interaction_ < 0.001). For males, the population attributable risk percentage using crude and age adjusted rates was calculated to be 18.9% and 19.8%, respectively. These respective percentages for females were 15.5% and 15.0%. There was no association between geographic remoteness and risk of SAH (Table 4).

**Table 4:** Incident rate ratios* (IRR) for subarachnoid hemorrhage (SAH) in New South Wales, Australia, 2001–2009.

	SAH rates		SAH fatal rates	
	IRR (95% CI)	*P*	IRR (95% CI)*	P*
**Sex**				
Males	Ref		Ref	
Females	1.33 (1.23–1.44)	<0.001	1.40 (1.24–1.59)	<0.001
**Age**				
20–49	Ref		Ref	
50–69	2.38 (2.06–2.74)		3.31 (2.69–4.09)	
70+	3.98 (3.46–4.57)	<0.001	14.89 (12.24–18.12)	<0.001
**Index of relative socio-economic disadvantage**				
Quintile 1 [low socio-economic status (SES)]	Ref		Ref	
Quintile 2	0.94 (0.82–1.07)		0.87 (0.68–1.12)	
Quintile 3	1.08 (0.97–1.21)		1.08 (0.88–1.33)	
Quintile 4	1.03 (0.92–1.15)		1.09 (0.88–1.34)	
Quintile 5 (high SES)	0.83 (0.74–0.92)	<0.001	0.81 (0.66–1.00)	0.003
**ARIA classification (geographical remoteness of residential location)**				
Highly accessible	Ref		Ref	
Accessible	0.99 (0.90–1.10)		1.01 (0.84–1.22)	
Moderately accessible	0.92 (0.80–1.06)		0.71 (0.53–0.95)	
Remote/very remote	1.19 (0.91–1.55)	0.37	1.26 (0.76–2.08)	0.08

*From negative binomial regression models adjusted for age, sex, and year.

**Figure 2:**
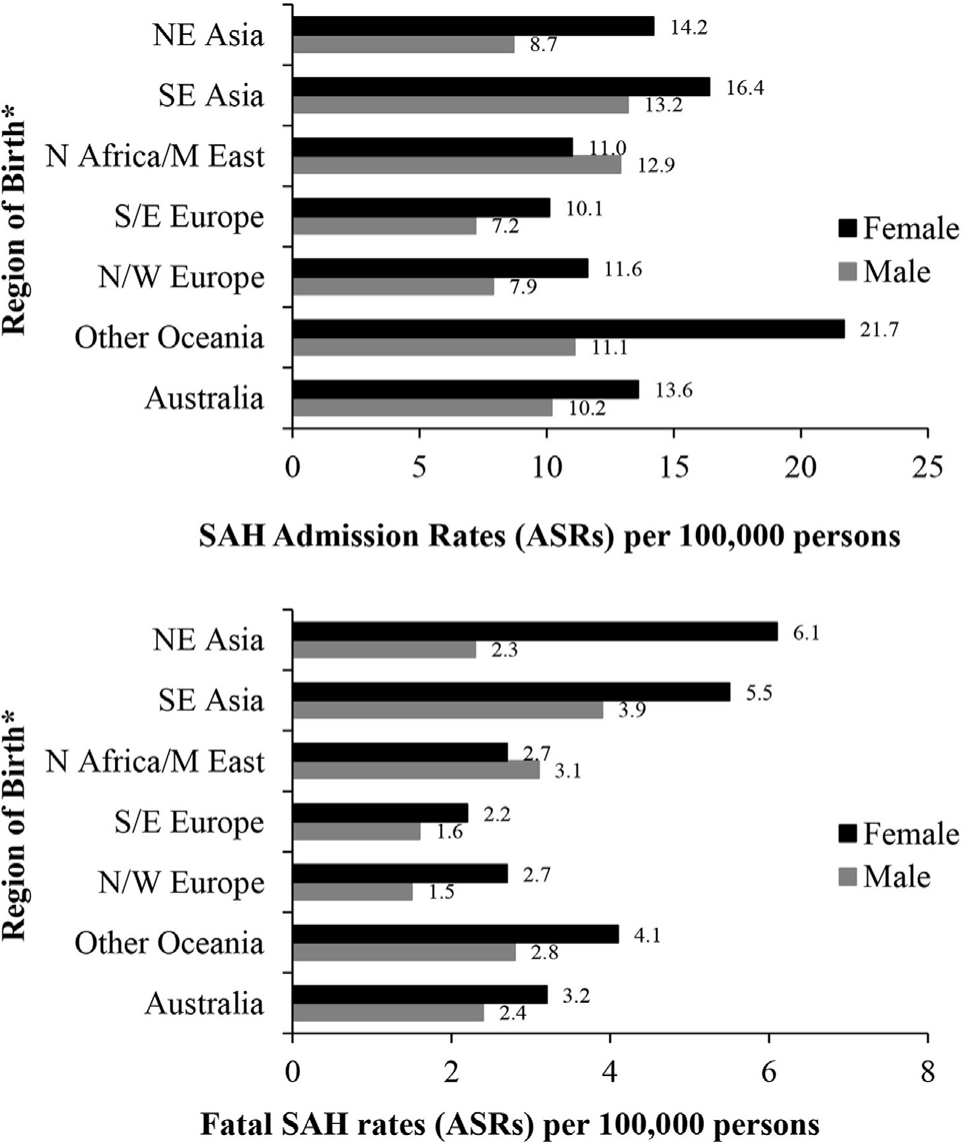
Age-standardized rates (ASR per 100,000) of subarachnoid hemorrhage (SAH) in New South Wales, Australia 2001–2009 by sex and region of birth. *Excludes resident population born in “other African,” “other Asian” nations and “The Americas” due to small numbers of SAH cases. Excludes 287 and 70 cases with missing country of birth data from analyses of admission rates and fatal SAH rates, respectively.

People born in South-East Asia and the Oceania region had a significantly higher risk of hospitalization for SAH, while the risk of fatal SAH was greater in South-East and North-East Asian born residents. People born in South-Eastern Europe had a lower risk of SAH. The increased risk of SAH admission and fatal SAH among females compared to males was seen in all groups except for those born in North Africa and the Middle East (Figure 2; Table 5).

**Table 5:** Age-standardized rates (ASR) per 100,000 of subarachnoid hemorrhage (SAH) admissions and fatal SAH in NSW in 2001–2009 by sex and region of birth.^a^

	Admission rates			Fatal rates		
Region of birth	Crude rate	ASR	95% CI	Crude rate	ASR	95% CI
**Males**						
Australia	10.9	10.2	9.6–10.8	2.7	2.4	2.1–2.7
Other Oceania	10.1	11.1	8.2–14.3	2.4	2.8	1.4–4.6
N/W Europe	10.3	7.9	6.6–9.3	2.4	1.5	1.0–2.1
S/E Europe	10.5	7.2	5.5–9.0	3.0	1.6	1.0–2.4
N Africa/M East	13.5	12.9	10.1–16.0	3.1	3.1	1.8–4.8
SE Asia	11.8	13.2	10.2–16.5	2.9	3.9	2.3–6.0
NE Asia	8.6	8.7	6.4–11.3	2.2	2.3	1.2–3.7
**Females**						
Australia	15.5	13.6	13.0–14.3	4.5	3.2	2.9–3.5
Other Oceania	20.9	21.7	17.9–25.8	3.7	4.1	2.5–6.1
N/W Europe	14.8	11.6	9.9–13.5	4.6	2.7	2.0–3.4
S/E Europe	14.6	10.1	8.1–12.3	5.1	2.2	1.6–2.9
N Africa/M East	11.4	11.0	8.3–14.1	2.9	2.7	1.5–4.3
SE Asia	13.7	16.4	13.5–19.7	4.0	5.5	3.8–7.6
NE Asia	12.7	14.2	11.4–17.3	5.3	6.1	4.3–8.1
**Both**						
Australia	13.3	12.0	11.6–12.4	3.7	2.8	2.7–3.0
Other Oceania	15.6	16.7	14.2–19.3	3.1	3.6	2.4–4.9
N/W Europe	12.5	9.7	8.6–10.8	3.5	2.1	1.7–2.6
S/E Europe	12.5	8.6	7.3–10.0	4.1	1.9	1.5–2.4
N Africa/M East	12.5	12.0	10.0–14.2	3.0	2.9	2.0–4.0
SE Asia	12.9	15.2	13.0–17.5	3.5	4.9	3.6–6.4
NE Asia	10.9	11.6	9.8–13.6	4.0	4.3	3.2–5.6

*^a^Excludes resident population born in “other African,” “other Asian” nations, and “The Americas” due to small numbers of SAH cases. Excludes 287 and 70 cases with missing country of birth data from analyses of admission rates and fatal SAH rates, respectively*.

### Sudden Deaths and Impact on Estimates of Attack Rates and Mortality

When limiting the study period to 2007 to include sudden deaths occurring outside hospital, 196 out of 3,979 SAH cases (4.9%) were identified from death data only. Crude estimates of SAH rates, increased by 1 per 100,000 person years when compared with admission rates for 2001–2009 (10.0 versus 9.0 for males and 13.9 versus 12.9 per 100,000 for females). The 30-day mortality rate was 35.5% with the addition of sudden death cases compared to 28.7% using admission data only for 2001–2009.

## Discussion

### Main Findings

This is the first large-scale, population-based study of SAH using data linkage reporting both admission rates and mortality outcomes to 1 year in Australia in the post-coiling era and one of the few recent studies internationally. The large number of SAH cases identified from within the same geographic location over a continuous period of 9 years is a major strength of the study.

We identified reductions in mortality *via* an analysis of fatal SAH using age-adjusted population-based rates and case fatality rates. The former metric determines the burden of fatal SAH by referencing mortality risk to population denominators, while case fatality measures the proportion of deaths among acute hospitalized SAH cases. These two measures were corroborative, providing evidence of falling mortality. Evidence for change in mortality mainly predates ISAT findings (1, 22, 23). In contrast, recent evidence for declining mortality is sparse. Two recent publications demonstrate declines in the USA (24) and England (25) for periods to 2010 and another two publications one each from the USA and UK for periods to 2008 (22, 26), while in-hospital mortality significantly declined from 2000 to 2015 in those select SAH patients requiring intensive care in Australia and New Zealand (27). Declines in fatal SAH rates were not apparent in those aged over 70 years, suggesting that their exposure to risk factors has not altered over time. Increased risk of rebleeding and large volume SAH due to anticoagulant use not amenable to neurointerventional care may explain why mortality has not decreased in elderly patients (28).

### Declining SAH Fatality

The reduction in case fatality in hospitalized SAH patients during a relatively short period of time corresponded with significant restructuring of health services in New South Wales, Australia. Interventional radiological and neurosurgical services were organized to facilitate uptake of evidence-based endovascular coiling in response to the ISAT findings, succeeding in delivering coiling to almost one-third of acute SAH patients, and this may have resulted in the mortality reductions reported in this state-wide population. We report mortality reductions from 2 days after admission, suggesting that this may be attributable to the corresponding wider uptake of time-critical treatments. During the study period, the uptake of coiling increased by eightfold from 3% at the beginning to around 30% of patients at the end. However, improved recognition and management of often fatal complications of SAH, such as hyponatremia and delayed cerebral ischemia, may have also contributed to improved outcomes. Co-ordinated funding enhancements to establish *de novo* units and enhance existing ones may have positively impacted on SAH outcomes by strengthening collaborations between stroke and other neurocritical care services and facilitating rapid transfer of patients.

By the end of the study period, 30-day case fatality was 22%. Reported 28- and 30-day SAH mortalities among hospitalized cases were 28% from other Australian state-wide cohorts in 1995–1998 (29) and in 2002 (30). The results underscore the potential impact that organized health service restructuring can have on acute SAH outcomes in the immediate aftermath of implementation. These findings invite inquiry into whether these improvements in outcomes have been sustained in the longer term as the state-wide neurosurgical network remains embedded within the health service jurisdiction.

In contrast to falling mortality, SAH admission rates remained static over time, suggesting that improvements in service delivery and not primary prevention may have underpinned survival gains. Some studies report declines (25, 31), and others report no change (14, 22–24, 26, 32) in SAH risk over time. In our 9-year study period, the risk of hospital admission for SAH was stable although declining rates in younger males may be attributable to reductions in smoking and alcohol intake in our community (33). An association between declining smoking rates and SAH incidence has been recently reported in Finland (31). The slight increase in admission rates seen in people older than 70 years of age may be due to adverse effects of antithrombotics or uncontrolled hypertension.

### Epidemiology of Hospitalized SAH

The burden of SAH in our community is inequitable, with the elderly and females more likely affected. This is consistent with previous findings (1, 2). Overall, we identified the crude risk of hospitalization for SAH as 12.9 and 9.0 per 100,000 in females and males, respectively. This compares to estimates of SAH incidence for both in- and out-of-hospital cases of 11.5 and 9.2 per 100,000 females and males, respectively, reported in a systematic review of 18 gold standard epidemiological studies providing sex-specific incidence rates (2). While the increased risk in women remains largely unexplained, propensity for greater aneurysmal formation and/or rupture has been suggested predominately due to hormonal factors, and specifically, changes in estradiol levels accompanying the menopause (34). Risk factors are implicated in mediating the risk in women to a greater extent than in men with a recent study suggesting that smoking exerts a greater risk for women than men (35). In our study, the preponderance of hospitalizations for females compared to males occurred from age 40 years onward. In other studies conducted outside Australia, the divergence of risk between females and males occurred in those older than 55 years, although variability in when women begin to realize their greater risk has been noted (1, 2). We may have been better able to detect an increased risk in women at a younger age due to greater power afforded by relatively large numbers of SAH (~1,000), although a recently reported study from Finland also utilizing administrative datasets found increased risk in women from the age of 55 years onward (31). The possibility that women in Australia are younger at age of onset cannot be discounted (32).

Geographic variation in SAH risk and mortality may belie underlying genetic predispositions (1); however, international comparisons between epidemiological studies are confounded by differences in selection criteria, case ascertainment, health service delivery, and the era during which studies were conducted. In contrast, a unique aspect of our analysis was the ability to assess SAH risk due to region of birth within the same geographical space and time and subject to the same standardized ascertainment across the health service. In this cohort, 30% were overseas born affording an ideal setting in which to explore variations in risk due to region of birth within the same time and health service. We compared risk between people born in seven global geographic regions. We identified female residents born in the Oceania region as having a particularly high risk of SAH; those born in North-East or South-East Asia were at a higher risk of fatal SAH. Lower risk was noted in people born in Southern and Eastern Europe. Of note, sex differences in SAH risk were not found in residents born in the Middle East/North Africa, while female preponderance was identified in all other subgroups. Heightened risk in females born in Oceania outside Australia, particularly relatively younger women, corroborates earlier findings (36). Differences in the prevalence of risk factors, particularly smoking and systolic blood pressure, or in the propensity to suffer ill-effects of smoking may explain ethnic variability in SAH (37, 38), although evidence remains inconclusive. A question deserving further exploration is whether the risks reported here apply to the specific immigrant communities in our population or would generalize to their compatriots living elsewhere or in their countries of origin.

For every 100 SAH admissions, the results suggest that 20 and 15 would be avoided in males and females, respectively, if the risk of SAH in our population equaled that of the least socio-economically disadvantaged. The association between SES and SAH incidence in Australasia has been reported previously with 166 cases pooled from three epidemiological studies carried out in Australia and New Zealand from 1995 to 2003 (39). These results suggest persistent inequalities in SAH burden, despite a universal health system.

### Strengths and Limitations

Our analyses were enhanced using data linkage providing a large number of SAH cases continuously accrued over several years. The method allowed cost-effective assessment of time trends in SAH epidemiology, which may not otherwise be feasible with resource-intensive gold standard case ascertainment. One potential limitation of the study is reliance on administrative data. While the accuracy of SAH coding varies, ICD-10 coding for hospitalized cases is often accurate (40). Positive predictive values for ICD-10 SAH coding of 85% (41), 91% (42, 43), 96.1% (44), and 97.6% (45) have been reported, and the sensitivity of ICD-10 case ascertainment reported in two studies was 88.9% (44) and 95% (41), indicating valid case ascertainment.

High levels of administrative coding accuracy in Australia have been reported. The positive predictive values of principal diagnoses are over 90 and ≥95% (46–50). High positive predictive values of 95% (46, 49) for principal stroke diagnostic codes have been reported in the Admitted Patient Data Collection. Researchers have also validated diagnostic coding of stroke mimics, including transient ischemic attack, migraine with hemiparesis, and hypoglycemia, and found that none of these cases were misclassified strokes, indicating that sensitivity of stroke coding is likely to be high (46). The accuracy of inter-rater reliability, sensitivity, and PPV of clinical coding against “expert” coding for cerebrovascular diseases, including SAH, were high in another Australian jurisdiction (kappa value 0.91, sensitivity = 89%, and positive predictive value = 93%, respectively) (51). The validity of SAH coding in the dataset utilized here is further supported by the comparability of findings to those reported in gold standard studies and in the similarity in the patterns of risk due to age and sex.

The main analyses relate only to SAH cases admitted to hospital and thus do not represent all cases of SAH in the population, necessarily excluding cases that died before reaching hospital. However, a sensitivity analysis utilizing cause of death mortality data for part of the study period showed that 5% of SAH die suddenly without reaching hospital and their inclusion minimally affected estimates of SAH risk, although case fatality rates increased by around 6% when accounting for cases of “sudden death.” However, limiting our main analyses to hospitalized cases is appropriate given our focus on examining the impact of health service delivery on SAH outcomes.

## Conclusion

Our study reports declining mortality risk in SAH corresponding to identifiable changes in health service delivery and evolving treatments such as coiling. Static SAH admission rates, except in young males, suggest that risk is difficult to ameliorate. Risk widely varied according to region of birth and SES. Addressing inequities in SAH risk and mortality may require the targeting of prevalent and modifiable risk factors to improve population outcomes.

## Ethics Statement

The study was carried out in accordance with the recommendations of the NSW Population and Health Services Research Ethics Committee and the Human Research Ethics Committee University of New South Wales. A consent waiver was granted for researchers to utilize linked routinely collected administrative data from which patient identifiers were removed.

## Author Notes

First published as a conference abstract at the Stroke Society of Australasia Annual Scientific Meeting, Sydney 2012.

## Author Contributions

JW contributed to study conception and design, acquisition of data, interpretation of analyses, drafting, revising, and final approval of manuscript, and accountability of work. BJ contributed to study design, acquisition of data, interpretation of analyses, revising and final approval of manuscript, and accountability. CG contributed to study design, acquisition of data, data analysis revising, and final approval of manuscript. MG contributed to study design, acquisition of data, interpretation of analyses, drafting, revising, and final approval of manuscript, and accountability of work.

## Conflict of Interest Statement

JW was Medical Co-Chair of Stroke Services NSW (2012–2014), an honorary leadership position within the NSW Ministry of Health tasked with executive decision making regarding stroke services. JW currently serves as a Board Member of the Bureau of Health Information, NSW Ministry of Health. All other authors declare no conflict of interest.
